# Spatio-Temporal Prediction for the Monitoring-Blind Area of Industrial Atmosphere Based on the Fusion Network

**DOI:** 10.3390/ijerph16203788

**Published:** 2019-10-09

**Authors:** Yu-ting Bai, Xiao-yi Wang, Qian Sun, Xue-bo Jin, Xiao-kai Wang, Ting-li Su, Jian-lei Kong

**Affiliations:** 1 School of Computer and Information Engineering, Beijing Technology and Business University, Beijing 100048, China; 2 Beijing Key Laboratory of Big Data Technology for Food Safety, Beijing Technology and Business University, Beijing 100048, China; 3 College of Physics and Electronic Engineering, Shanxi University, Taiyuan 030006, China

**Keywords:** time series prediction, unknown inference, atmospheric quality, neural network

## Abstract

The monitoring-blind area exists in the industrial park because of private interest and limited administrative power. As the atmospheric quality in the blind area impacts the environment management seriously, the prediction and inference of the blind area is explored in this paper. Firstly, the fusion network framework was designed for the solution of “Circumjacent Monitoring-Blind Area Inference”. In the fusion network, the nonlinear autoregressive network was set up for the time series prediction of circumjacent points, and the full connection layer was built for the nonlinear relation fitting of multiple points. Secondly, the physical structure and learning method was studied for the sub-elements in the fusion network. Thirdly, the spatio-temporal prediction algorithm was proposed based on the network for the blind area monitoring problem. Finally, the experiment was conducted with the practical monitoring data in an industrial park in Hebei Province, China. The results show that the solution is feasible for the blind area analysis in the view of spatial and temporal dimensions.

## 1. Introduction

Atmospheric pollution has been a severe problem since the end of the last century. It is still a challenge especially in developing countries, where the industrial park is a universal mode of production and has been the primary pollution source [1]. It is vital to manage and control the industrial emissions for the atmospheric environment. In the management, an essential task is to monitor the factories which are located in the industrial park. The direct solution is arranging the monitoring stations in every factory. However, the solution becomes impractical according to our survey of some provinces in China. Under the insufficient administration authority, the monitoring station inside the factory cannot be established because the monitoring equipment may be impacted artificially, or even destroyed. Some monitoring-blind areas are formed in these situations. In this case, an indirect solution should be explored. It is also found in the survey that the monitoring grid scheme has been applied in China where sensors are arranged at the cross point of the rectangular grid. As such, existing monitoring sensors can be the auxiliary for the analysis and inference of the monitoring-blind area. The monitoring-blind area discussed in the paper can be defined as a location in the industrial park that it is a critical monitoring and management point for the atmospheric pollution, but cannot be monitored directly with the inner equipment. The main issue in this paper is the analysis and inference of the monitoring-blind area with the circumjacent sensors.

The issue above belongs to the spatial analysis of the atmospheric environment. The common spatial analysis tool mainly includes the Gaussian puff model and plume model [2,3]. The models describe the relations of the gas source concentration, monitoring location, wind speed, wind direction, and other meteorological factors. They have been applied in the evaluation of the air quality and pollutant dispersion [4,5]. The models are built based on the mathematical mechanism, in which the parameters are always given with artificial experience. Besides, the model is challenging to build because of the error accumulation in physical applications. As such, the data-driven methods become the optional solutions for spatial analysis of the atmospheric environment.

Apart from the spatial analysis, there is also a demand for prediction in atmospheric environment management. As well as the primary real-time monitoring data, the prediction information plays an essential role in the trend analysis and further prevention. Some precautionary measures can be conducted based on the prediction to lower the environmental risks and save the management cost. In the studies of time series prediction, the statistical methods and machine learning models are the mainstream. Moreover, machine learning has attracted more attention because of its strong ability in nonlinear regression with data features.

It can be found that the data-driven method of the spatial analysis should be explored, overcoming the shortcomings of the traditional mechanism model. Meanwhile, a sufficient time series prediction model should be established. Moreover, how to merge the two functions in a unified model is a challenge. A fusion network framework is proposed catering to the duplex prediction demands in the spatial and temporal dimensions. Different networks are studied and designed for the multipoint inference and time series prediction. In the network, the data feature is focused instead on the complicated spatial mechanisms in the traditional Gaussian models. The method is verified with the practical data from an industrial park of Hebei Province in China. The results show that the solution is feasible for the spatio-temporal prediction issue.

The rest of this paper is organized as follows. Section 2 introduces the related studies, including the time series prediction methods and the spatial analysis methods for the atmospheric environment. In Section 3, the main fusion model is proposed, and the physical networks are designed for different demands. Experiments are conducted in Section 4, and the results are discussed in Section 5. Finally, the study of the paper is concluded in Section 6.

## 2. Related Works

In the paper, the atmospheric quality in the industrial park is analyzed from the dimensions of space and time. For the temporal dimension, the main task is the modeling of time series and the prediction of the data trend. For the spatial dimension, the distribution of the atmospheric pollution sources should be analyzed. Following this, the related works are collected and arranged from the two parts.

### 2.1. Time Series Prediction Methods

The time series analysis methods have been studied and applied widely, in which two main categories of methods have become the mainstream, including the statistical and machine learning methods.

The statistical methods mainly describe the internal features in time series with the mathematical analysis. The typical statistic model consists of the autocorrelation function and hypothetical noise. The auto regression (AR) model [6] and moving average (MA) model [7] are the basic statistical models. They describe the time series changes with data themselves. For the AR model, the subsequent data in the next steps are indicated with the linear combination of the previous variables. The sliding window is introduced in the MA model, and it allows the data slot to reflect the time series features dynamically. For the precise modeling of time series in the nonstationary mode, some improved modes are introduced in which the autoregressive moving average (ARMA) [8] and autoregressive integrated moving average (ARIMA) [9] have been the typical models. ARIMA combines the AR, MA, and calculus of differences, and it has been applied in many prediction issues of the time series [10,11].

The mathematical basis of the statistic models above is the hypothesis of the stationary degree of the time series. Although the operations are taken in the stationary transform, the prediction performance declines in the nonlinear long-term prediction. With modern data-driven thought, machine learning has been an important tool in data classification and fitting. The prediction methods based on machine learning developed from the traditional shallow models to the popular deep learning methods. Their common ground is the modeling of data in the thought of a black box. They are distinct in the model structure complexity and the calculation scale. The shallow models mainly include the back-propagation neural network (BP), support vector machine (SVM), nonlinear autoregressive neural network (NAR), and Bayes network [12]. In deep learning, the recurrent neural network (RNN) [13] is the main tool of time series prediction. The improvements of RNN are proposed, such as the multidimensional RNN [14] and the bidirectional RNN [15]. Long short-term memory networks [16] represent the improvements of RNN. It mainly solves the long-term dependency problem. Although the deep networks usually perform better than traditional networks, their structures are more complex, and they need more training time and computing resources.

Scholars have explored the machine learning methods in the prediction of the atmospheric environment. The BP network is the typical and prevalent tool in studies. The underlying network and the improvements combined with other optimization methods have been applied in different atmospheric indexes [17,18,19]. Moreover, machine learning methods fused with other numeric analysis methods were also tested. Xin [20] introduced the genetic algorithm to the Bayes network and built the prediction model of air quality, which was applied in the concentration prediction at Qingdao of Shandong Province in China. Yu et al. [21] considered the different diffusion models to predict the concentration of Particulate Matter 2.5 (PM 2.5) in the time scale of an hour. Liu et al. [22] predicted the concentration of PM 2.5 with the extreme learning machine, which is a miniaturized network. Wang et al. [23] combined ARIMA with SVM to design a hybrid Garch model that was applied in the PM 2.5 prediction. García Nieto et al. [24] explored the different machine learning methods in the air quality modeling, including the particle swarm optimization with SVM, multilayer perception network, and model tree. It can be seen from the explorations that an appropriate model should be selected and built considering the nonlinear fitting ability as well as the network complexity. A balance of performance and calculation scale can help the practical application.

### 2.2. Spatial Analysis of Atmospheric Environment

The flowability is the prominent characteristic of the atmospheric elements. The circumjacent environment influences the atmospheric quality at a point in the action of airflow. Then the gas diffusion under the flowability becomes a research spot. The typical gas diffusion models can be divided into two categories, the computational fluid dynamics model (CFD) [25,26] and the probability model. The CFD model is accurate and professional for gas diffusion analysis. It involves a specialized mechanism based on the hydromechanics, which leads to the professional requirement and the complex calculation process. The probability model mainly realizes the posterior probability statistics of a specific point in time with the prior probability. The diffusion information is estimated with the probability value. The standard probability model includes the Gaussian puff model [2], Gaussian plume model [3], state-space model [27], and hidden Markov model [28]. The Gaussian model performs well in the simple model form and small calculation scale. It has been applied widely in the distribution analysis of the atmospheric environment. Zhou et al. [29] analyzed the air quality in Chengdu, in which the diffusion diagram of the point source was built in the Gaussian three-dimensional space. In [30], the simulation program was built in MATLAB Available online: https://ww2.mathworks.cn/products/matlab.html (accessed on 6 October 2019). based on the basic Gaussian model. The iterative computation in the software updated the parameters, and the air quality in Hebei Province and Tianjin was tested with the system.

In the application of the Gaussian model, the ideal hypothesis is the basis of analysis. For example, the concentration should be the normal distribution in the horizontal and vertical axes, and the source should be continuous and homogeneous where the inner humidity and temperature are evenly distributed, the diffusion coefficient of the atmosphere is isotropic, and so on. The hypothesis leads to the problematic fitting and the result drifting in some complex atmospheric environments. Then the tools which differ from the mechanism model may be effective. The data-driven methods can be explored in the spatial analysis based on the monitoring information at multiple points.

## 3. Fusion Network of Spatio-Temporal Prediction

### 3.1. Problem Description

With the survey in some industrial parks in different districts of China, it is impractical to arrange the monitoring points in the factories, as some factories may reject the installation, or even destroy the monitoring equipment, to avoid supervision. Some may affect the monitoring environment artificially with air blowers so that the monitoring data are not accurate. The problems cannot be solved entirely because of the administrative power division in different organizations. As such, a technique is explored in this paper under the existing management policy.

**Figure 1:**
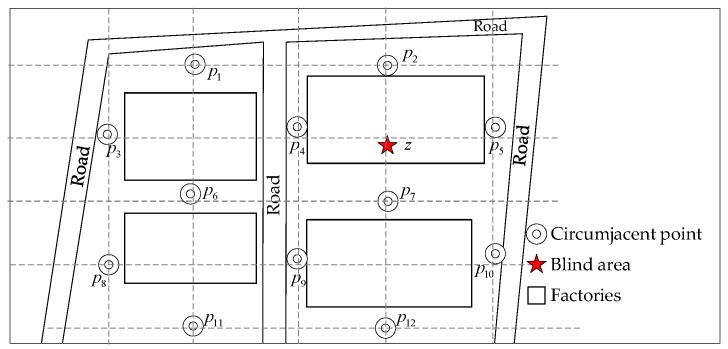
Layout of monitoring points and blind area.

The fundamental thought behind the solution is to determine the atmospheric quality data in a factory with the monitoring information of the circumjacent points. The solution is called “Circumjacent Monitoring-Blind Area Inference”. The layout of the monitoring points is shown in Figure 1. Some monitoring points are selected as the reference according to the existing practical monitoring grid. Then the mapping relation between the blind area and the circumjacent data is regressed with the neural network. The circumjacent monitoring points indicated as $<p_{1},p_{2},\cdots ,p_{m}>$, and m are the number of points. The expected monitoring point in the blind area is z. There are data series with the same sampling interval Δt and monitoring duration T, and these series are indicated as $D^{pi}$. The nonlinear relation f is the mapping between atmospheric concentration data $D^{z}$ and $<D^{p1},D^{p2},\cdots ,D^{pm}>$.

The relation function f describes the mapping of different locations at the same time point. The function f will be modeled with the data-driven method, in which the data feature in the different locations is extracted. As mentioned in the introduction, the future trend also plays an important role in advanced prevention. Following this, the time series features also need to be extracted. The trend function $g_{pi}(i=1,2,\cdots ,m)$ will be explored with the neural network, which is fit for the time series. Based on the data models, the current atmospheric concentration of a blind area can be determined with f, and the future concentration can be obtained when the prediction data of circumjacent points are obtained with $g_{pi}$. The functions will be explored and modeled in the following sections.

### 3.2. Fusion Network Framework

**Figure 2:**
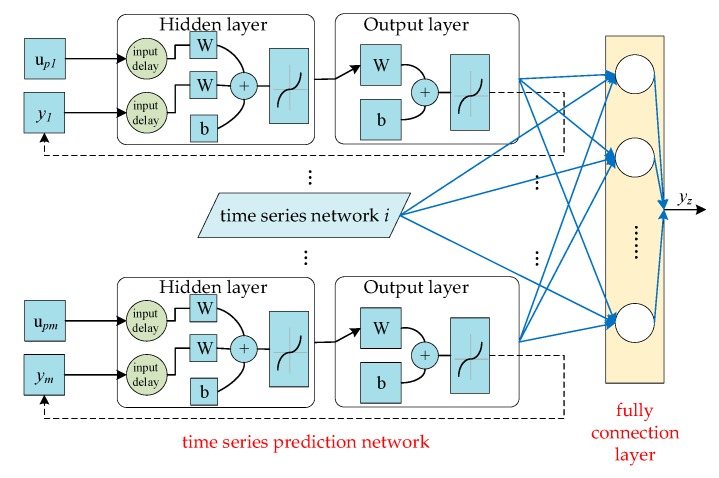
Structure of fusion network for “Circumjacent Monitoring-Blind Area Inference”.

In the “Circumjacent Monitoring-Blind Area Inference” solution, there are two tasks to conduct, one of which is the time series prediction of each circumjacent point, and the other one is the inference of the blind area with the circumjacent information. Then, a fusion network framework is designed, as shown in Figure 2.

In Figure 2, the fusion network consists of two main parts marked with the captions in red. Firstly, for the time series prediction network, the nonlinear autoregressive network with exogenous inputs (NARX) [31] is set as the primary structure for each point. The NARX can model the target variable in the time dimension based on the feedback structure. Meanwhile, it considers the related variables which affect the target variable potentially. The output of the variable is the exogenous inputs in the NARX. For the NARX in Figure 2, $u_{pi}$ is the index related to the target index in the circumjacent point, $y_{i}$ is the output of the target index in each point, W is the weight matrix, and b is the bias. The network variables will be explained in Section 3.3. Secondly, the data in the blind area is determined with a structure of full connection layers. The mapping relation of the different monitoring points is modeled with the full connection layer. The prediction results of the target variable in each circumjacent point are imported into the layer to be integrated as the final output $y_{z}$ of blind area data.

For the fusion network, the outputs of the NARX are imported into the fully connected layer. The two parts are the vital primary models. The time series prediction model based on the NARX and the spatial prediction model based on the full connection layer will be studied respectively in Section 3.3 and Section 3.4. Then the algorithm of “Circumjacent Monitoring-Blind Area Inference” can be concluded finally.

### 3.3. Time Series Prediction Model Based on NARX

**Figure 3:**
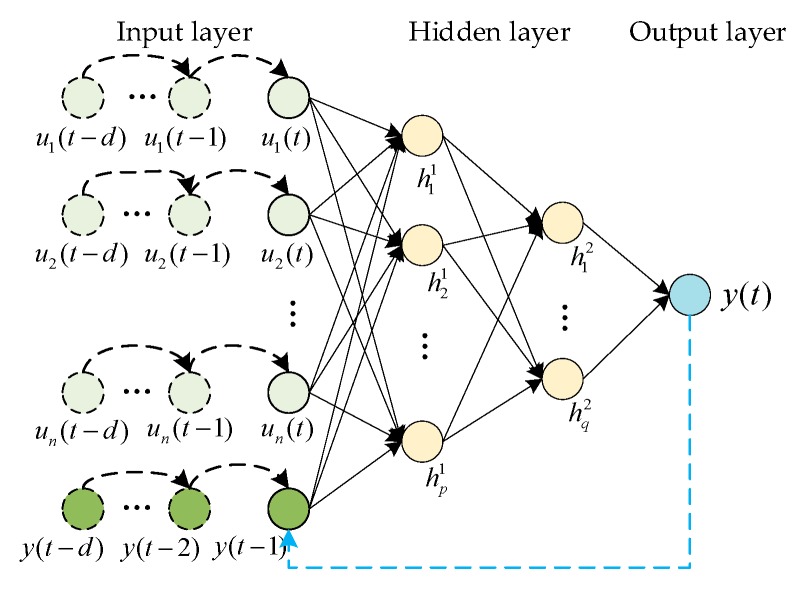
The network for the time series prediction based on the NARX structure.

The neural network based on the NARX structure is built for the time series prediction of the circumjacent point. On the one hand, the target variables should be predicted over time. On the other hand, the influence of the related variables should be considered. For example, the SO_2_ concentration is impacted by other meteorological variables such as the wind and humidity. Based on the two demands, the time series model is designed, as shown in Figure 3.

For the time series network in Figure 3, there are three kinds of layers, including the input, hidden, and output. The hidden layers can be extended to multiple layers according to the nonlinear complexity degree. As an empirical design, the hidden layers are usually one to four in shallow learning. The layers should not be too many, avoiding the divergency in the network training. The two hidden layers in Figure 3 are the conceptual representation, which can be another layer number in practice. In the following discussion, the network is introduced as a typical unit for a circumjacent point, so the number of the points is omitted. The inputs include the target variable y and the related variables $u_{j}(1\leq j\leq n)$, where n is the number of related variables. The network can be expressed as

(1) $$y(t)=f\left(y(t-1),y(t-2),\cdots ,y(t-d),u_{j}(t),u_{j}(t-1),\cdots ,u_{j}(t-d)\right)$$

where d is the input delay, which means the number of previous time steps before the present moment.

The relation between the input and hidden layer is:

(2) $$h_{k}^{1}=f(\sum_{j=1}^{n}\omega_{jk}u_{j}+a_{1})$$

(3) $$h_{r}^{2}=f(\sum_{k=1}^{p}\omega_{kr}h_{k}^{1}+a_{2})$$

where $h_{k}^{1}$ and $h_{r}^{2}$ are the neurons in hidden layers, k=1,2,⋯,p;r=1,2,⋯,q,p and q are the number of neurons, f is the activation function of the hidden layer, ω is the connection weight between the adjacent neurons, and $a_{1}$ and $a_{2}$ are the threshold value of the hidden layers. The output is derived from the hidden layer:

(4) $$y=f(\sum_{r=1}^{q}h_{r}^{2}\omega_{r}+b)$$

where b is the threshold of the output layer.

The training method should be studied based on the physical network above. Similar to the shallow networks, the time series network based on the NARX can be trained with the back-propagation mechanism. The error of the network between the prediction output and expected output is

(5) $$e=\frac{1}{2}{\left(\hat{y}-y\right)}^{2}$$

where e is the error, y is the prediction output, and $\hat{y}$ is the expected output from the measurements.

The connection weights $\omega_{jk},\omega_{kr},\omega_{r},a_{1},a_{2},b$ are adjusted with the errors until the global error or the training iterations reach the preset value. Based on the back-propagation algorithm, the weights are obtained as

(6) $$\{\begin{array}{l} \omega_{jk}=\omega_{jk}+\eta h_{k}^{1}(1-h_{k}^{1})u_{j}\omega_{kr}e\\ \omega_{kr}=\omega_{kr}+\eta h_{r}^{2}(1-h_{r}^{2})h_{k}^{1}\omega_{r}e\\ \omega_{r}=\omega_{r}+\eta h_{r}^{2}e\\ a_{1}=a_{1}+\eta h_{k}^{1}(1-h_{k}^{1})\omega_{kr}e\\ a_{2}=a_{2}+\eta h_{r}^{2}(1-h_{r}^{2})\omega_{r}e\\ b=b+e\\ E=\frac{1}{2r}\sum_{r=1}^{q}{\left(\hat{y}-y\right)}^{2} \end{array}$$

where η is the learning rate and E is the global error of the network.

For every circumjacent monitoring point, a separate network can be trained based on the structure and learning method above. The networks run with the same time steps to provide the basic prediction information. Then the predicted results are set as the inputs of the full connection layer in the spatial inference model.

### 3.4. Spatial Inference Model

In the fusion network shown in Figure 2, the monitoring data of the blind area is obtained based on the structure of the full connection layer. For the full connection layer, its inputs are the outputs of the time series networks of the circumjacent points, namely $Y={\left(y_{1},y_{2},\cdots ,y_{m}\right)}^{T}$. The final output is o and the expected output is s, which is from the temporary monitoring equipment. The weight from the input layer to connection layer is $V=(v_{1},v_{2},\cdots ,v_{i},\cdots ,v_{m})$, and the weight from the connection layer to the final output layer is $W=(w_{1},w_{2},\cdots ,w_{t},\cdots ,w_{K})$.

The model of the full connection layer can be expressed as

(7) $$o=f\left(\sum_{t=1}^{K}w_{t}f\left(\sum_{i=1}^{m}v_{i}y_{j}\right)\right)$$

where f is the transfer function and can be selected as the unipolar sigmoid function:

(8) $$f(x)=1/(1+e^{-x})$$

where f(x) has the characteristics of continuous and differentiable, and

(9) f’(x)=f(x)[1−f(x)]

There is an output error E when the network output is not equal to the expected output.

(10) $$E={\left(s-o\right)}^{2}/2=\sum_{t=1}^{K}{\left(s_{t}-o_{t}\right)}^{2}/2$$

The error can be derived based on the formula above, and

(11) $$E=\frac{1}{2}\sum_{t=1}^{K}[s_{t}-f(\sum_{i=1}^{m}w_{it}f(\sum_{j=1}^{n}v_{ji}y_{j})){]}^{2}$$

It can be seen that the error is a function of the weight of each layer. We can adjust the output error by adjusting the weight of each layer, and we make the output error tend to zero eventually.

(12) $$\Delta w_{it}=-\eta \frac{\partial E}{\partial w_{it}}$$

(13) $$\Delta v_{ji}=-\eta \frac{\partial E}{\partial v_{ji}}$$

where η is the learning rate, 0<η<1. Correspondingly, the final layer Formula (12) and the hidden layer Formula (13) can be rewritten as:

(14) $$\Delta w_{it}=-\eta \frac{\partial E}{\partial w_{it}}=-\eta \frac{\partial E}{\partial net_{t}}\frac{\partial net_{t}}{\partial w_{ij}}$$

(15) $$\Delta v_{ji}=-\eta \frac{\partial E}{\partial v_{ji}}=-\eta \frac{\partial E}{\partial net_{i}}\frac{\partial net_{i}}{\partial v_{ji}}$$

Otherwise, we assume that the final output layer and the hidden layer of the error signal are:

(16) $$\delta_{t}^{o}=-\frac{\partial E}{\partial net_{t}}=-\frac{\partial E}{\partial o_{t}}f’(net_{t})$$

(17) $$\delta_{i}^{y}=-\frac{\partial E}{\partial net_{i}}=-\frac{\partial E}{\partial y_{i}}f’(net_{i})$$

The formulas constitute the simultaneous equations, one group is Formulas (9)−(11), (14) and (16) and the other one is Formulas (9)−(11), (15) and (17). The weight adjustment is obtained finally by solving the equations, and they are

(18) $$\Delta w_{it}=\eta \delta_{t}^{o}y_{i}=\eta (s_{t}-o_{t})o_{t}(1-o_{t})y_{i}$$

(19) $$\Delta v_{jt}=\eta (\sum_{t=1}^{K}\delta_{t}^{o}w_{it})y_{i}(1-y_{i})$$

The full connection layer in the fusion network can be trained with the weight adjustment method above. Then the inference result of the blind area can be obtained. Besides, the blind area data can also be predicted because it is of the same time step with the first time series network. When the future values in the next steps are predicted by the front networks, the blind area data update synchronously to obtain the future trend.

### 3.5. Spatio-Temporal Prediction Algorithm

**Figure 4:**
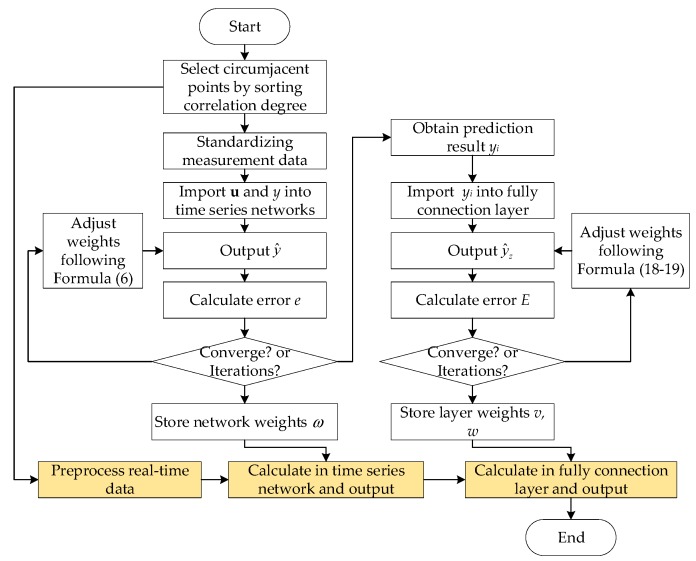
The spatio-temporal prediction algorithm flow.

The spatio-temporal prediction algorithm is concluded here based on the fusion network framework and the physical networks. The algorithm can guide the employment of the monitoring data to obtain the inference and prediction results of the blind area. The flow of the algorithm is shown in Figure 4.

For the neural network, two operations should be taken to apply it, of which one is the training process, and the other is the test process. Then, the algorithm consists of two parts based on the training and testing of the network. The physical flow of the prediction algorithm is as follows:

(1) Training of the time series network;

(a) The location of the monitoring-blind area is confirmed. The monitoring points around it are set as alternatives. The correlation degree between the target and alternative points can be obtained to select the final circumjacent points;

(b) The measurement data are collected at each circumjacent point. Meanwhile, the temporary monitoring equipment should be settled at the blind area for the modeling in the name of research;

(c) The historical monitoring data are imported into the time series networks. For the variable data in different units, they are preprocessed with (0,1) standardization. The networks can output the initial prediction results;

(d) The errors between the outputs and the expected values are calculated and validated;

(e) If the error is lower than the present value, or the iteration times reach the preset parameter, the training can be over. Then the final weights are stored. Otherwise, the weights should be adjusted following Formula (6) in Section 3.3.

(2) Training of the full connection layer;

(f) When the training of the time series network is over, the outputs of the circumjacent points are imported into the full connection layer;

(g) The training process of the full connection layer is similar to the training of the time series network from step (c) to (e), following the weight adjustment in Formulas (18) and (19).

(3) Prediction and inference of real-time data;

(h) When the whole network is trained, it can be applied to predict the real-time data. The calculation process is shown in Figure 4 with blocks in yellow.

The fusion network is established with the time series network and the full connection layer. The structure and training methods of the network are studied. Finally, the spatio-temporal prediction algorithm is proposed, which can be the reference solution for the monitoring problem in some blind areas in industrial parks.

## 4. Experiment and Result

### 4.1. Experiment Data and Setting

The experiment was designed and conducted based on the practical monitoring of an industrial park in Hebei Province, China. As mentioned in Section 3.1, it is usually tricky to set up the monitoring equipment in the factories. Moreover, the government has established a monitoring system in which the monitoring stations are located at the cross point of the rectangular grid. The sparse layout makes it challenging to analyze the atmosphere distribution with the traditional Gaussian mechanism models. The data-driven method proposed is applied in the practical monitoring problem.

**Figure 5:**
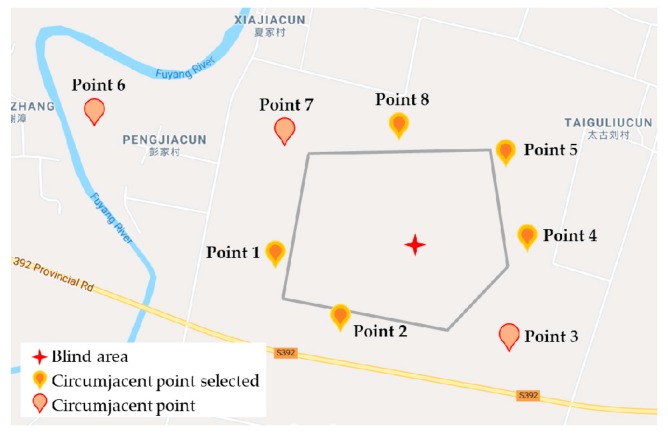
Layout of the monitoring grid and the points in the selected industrial park.

The experiment area is shown in Figure 5, in which there are eight monitoring stations settled by the government, and the location marked with a red star is the monitoring-blind area to be analyzed. For the research aim, temporal monitoring equipment was arranged in the factory under negotiation. The temporal equipment was only for the research of the modeling, and it cannot be used in routine monitoring. The model built in this paper is expected to replace the temporal equipment with “Circumjacent Monitoring-Blind Area Inference”.

**Figure 6:**
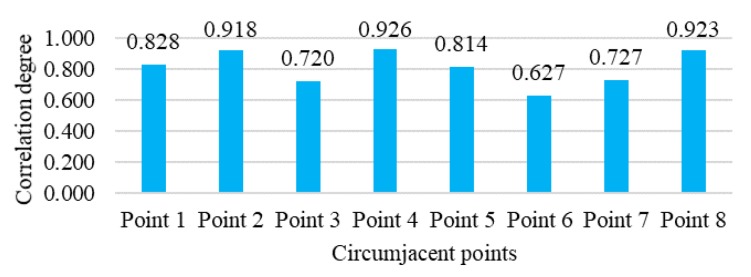
Correlation degrees of different circumjacent points with the blind area.

Limited by the network size and calculation load, the most related circumjacent points should be selected. The correlation degree of the points was calculated with the data from 14 days, shown in Figure 6. Then five points were selected with the correlation degree, including Points 4, 8, 2, 1, and 5. The screening of the related points can reduce the network complexity and eliminate the effect of the sparse points from the spatial view. The selected points are shown in Figure 5, with a different mark among all the points.

For the monitoring points, the atmospheric quality data were collected with the automation monitoring system. The atmospheric quality indexes included SO_2_ concentration, temperature, humidity, wind speed, and wind direction. The data in the experiment were monitored in January 2017. The monitoring interval was 30 min. The total number of data was 1488 in the experiment.

For the modeling of the neural network, the data were usually divided into three parts: training, validation, and testing. The number of data in the training and validation was 1100 sets, in which the validation part was selected with the hold-out method. The others were divided into two groups for the test (one group was 288 sets, and the other was 100 sets). For validation and testing, some error indicators were introduced to evaluate the network’s training. The indicators included the mean absolute error (MAE) and root mean squared error (RMSE).

For the networks in the experiment, five time series networks were built according to the number of circumjacent points. In the time series network, the inputs included the atmospheric quality indexes. The indexes were with the different units, and they were normalized to (0,1) when importing into the network. The output of the network was reversely normalized to restore the data in the original unit. The parameters of the network are listed in Table 1. There were five time series networks (equal to the number of circumjacent points) in the fusion network, and the parameters in the table are for each network. For each time series network, the inputs included the target index SO_2_ and correlated indexes of temperature, humidity, wind speed, and wind direction. Then the number of inputs was five. The numbers of neurons in hidden layers were ensured with the empiric value according to the number of inputs and outputs.

Some related models are set as contrast methods:

(1) The basic neural network (BP network) was built, in which the SO_2_ concentration of five circumjacent points was imported into BP network, and the output was the blind area data. This method is abbreviated to BP;

(2) In the same fusion framework of this paper, the time series network was replaced with ARIMA to realize the prediction function. The prediction results of SO_2_ concentration in five points based on ARIMA were imported into the full connection layer. This method is abbreviated to ARIMA-FC;

(3) In the same fusion framework, the full connection layer was replaced with the classical fusion method of weighted sum. The prediction outputs of five points based on NARX were summed directly with the trial weights. This method is abbreviated to NARX-WS.

Our method is abbreviated to NARX-FC. In the experiment, each method was adjusted to obtain the relatively optimal results.

### 4.2. Experiment Result

The fusion network proposed in this paper was trained and validated in the experiment. For the validation, three groups of the data were divided based on the hold-out method to calculate the output error. For the intuitive comparison with the test results, the validation result errors were reversely normalized from (0,1), and the errors are listed in Table 2. The errors in different subsets were approximate and within the accepted level. Then, the trained network can be regarded as available without divergency and overfitting.

**Figure 7:**
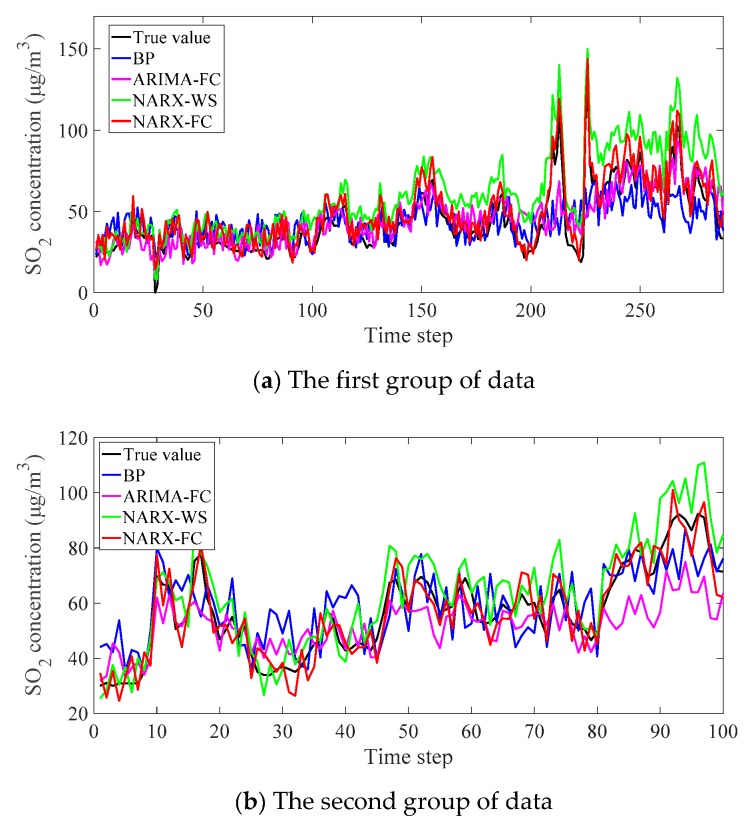
Prediction results of SO_2_ concentration.

When the network was established, two groups of the data were tested with the proposed method and contrast methods. In the first group, 288 sets of data were predicted. The second group was 100 sets. The results are shown in Figure 7 in which the concentration of SO_2_ is predicted with the time step delays. The input delay means the number of historical data used, which was 24 in the experiment. The output delay means the time steps to be predicted, and it was six in the test. That is, 24 sets of the monitoring data were used to predict the future data in the next six time steps. The data were used forward circularly. In Figure 7, the reference true value and the prediction results of various methods are presented with lines in different colors.

In the prediction results of the blind area, the methods show different performance. For the methods of BP and ARIMA-FC, they cannot trace the trend closely, especially in the apparent fluctuation (like the 210–230 steps in the first group). The other methods based on the NARX perform better in the trend tracing. The drifting of NARX-WS is severe. The proposed method NARX-FC performs reliably in general.

**Figure 8:**
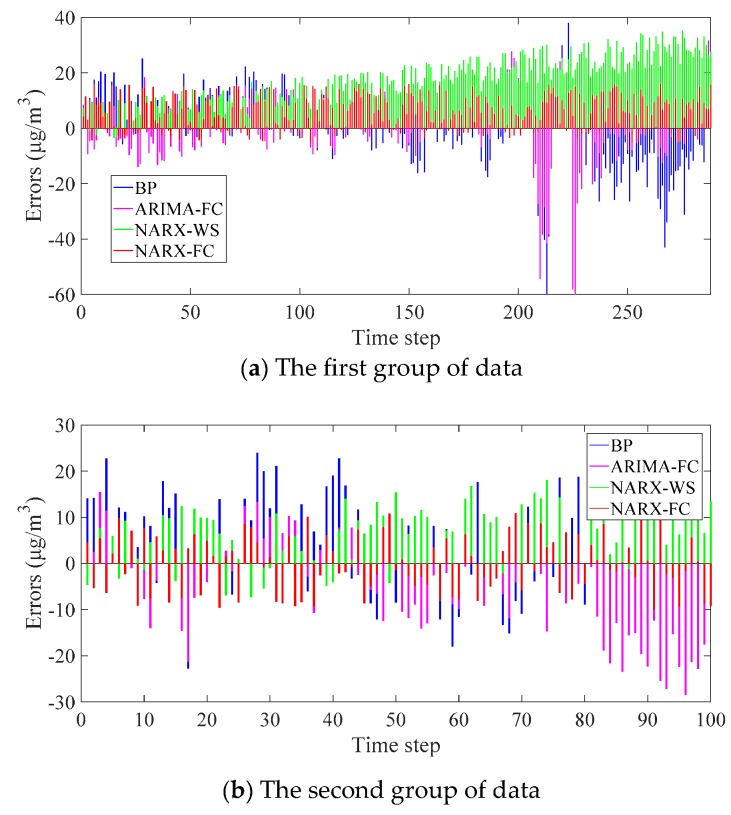
Errors of the prediction results in different methods.

To evaluate the different methods, the errors are calculated and shown in Figure 8. The error indicators MAE and RMSE are listed in Table 3.

It can be found from the error indicators in Table 2 and Table 3 that the errors in validation and testing are on the similar level. It proves that the network is steady in the experiment. The exhibition of the errors can explain the different performance of the methods. Similar to the trend in Figure 7, the errors of BP and ARIMA-FC are large in the data fluctuation. The errors of NARX-WS increase gradually, and the phenomenon is in accord with the drifting. The errors of NARX-FC are generally stable, and they are smaller than others. The performance can also be seen from the error indicators. For MAE, NARX-FC performs better than the others. MAE of results in NARX-WS is the largest in the first group, while BP and ARIMA-FC are similar. While MAE of BP is the largest in the second group of data. RMSE reflects the overall closeness of the results to the average value. It can indicate the stability of the prediction methods. The sort of RMSE in different methods is similar to the trend of MAE, and the proposed method NARX-FC is more stable than the others in the prediction.

## 5. Discussion

An indirect solution is designed to analyze the atmospheric quality data in a monitoring-blind area with the known circumjacent information. The method is firstly discussed with the experiment results. (1) For the inference of the monitoring-blind point, the external data feature is utilized in the data-driven solution. The solution can analyze the numerical relation without the calculation of spatial information such as the distance, layout, and airflow. It is realized with a full connection layer of which the performance is compared with the common fusion method of the weighted sum in NARX-WS. The weighted sum method realizes only the linear fusion of the circumjacent points, and it leads to the result drifting. The full connection layer is a feasible method for the nonlinear fitting with a controllable network size. (2) For the prediction of the monitoring-blind area, the NARX network performs better than the traditional statistical methods like ARIMA. The NARX network belongs to the shallow network of which the structure is relatively simple with the appropriate calculation scale.

From the theoretical view, the method belongs to the combination neural network. The fusion framework is relatively simple, but the experiment proves that it is valid for the analysis of the monitoring-blind area. The method is an exploration for the multiple functions of the network. More functions of the networks, such as the prediction, regression, classification, and optimization [32], can be combined for a target issue. Meanwhile, from the application view, the spatial and temporal analysis is combined for the industrial atmospheric management. In the modern data era, other problems try to be solved with the data-driven solution based on the improvement and combination of the network.

The method proposed in the paper provides a data-driven solution with the fusion network. The elements in the existing network are relatively limited, which is influenced by the network size and calculation load. In the ideal hypothesis, the more correlated the variables, the stronger the network fitting ability. In future research of the atmospheric management, other spatial variables can be set as the network inputs to enhance the inference reliability. Meanwhile, the increase of variables needs high network performance. Then, the improvement of the shallow network can be explored, as well as deep neural networks.

## 6. Conclusions

In general, a practical problem in industrial atmospheric management is studied. For the problem, some factories cannot be monitored directly due to private interest and administrative power. As such, the indirect solution of “Circumjacent Monitoring-Blind Area Inference” is proposed with a fusion network. In the fusion framework, the time series and fully connected networks are developed for prediction and nonlinear fitting. It is an improved and synthetic application of the neural network, which provides a data-driven solution for the practical interdisciplinary issue. In future work, more correlated variables can be introduced for a comprehensive model in the atmospheric analysis. As well as this, other data-driven methods can be studied in the spatial and temporal framework to support management and decision making.

## Figures and Tables

**Table 1:** Data used in the experiment.

Network	Time Series Network (for Each)	Full Connection Layer
Number of training times	1100	1100
Learning rate	0.01	0.01
Convergence error	0.002	0.002
Input delay	1:24	\
Output delay	1:6	\
Number of inputs	5	5
Number of outputs	1	1
Number of first hidden neurons	8	7
Number of second hidden neurons	4	\

**Table 2:** Error indicator of the validation results. Mean absolute error, MAE; root mean squared error, RMSE.

Error Indicator	Validation Subset 1	Validation Subset 2	Validation Subset 3
MAE	4.5683	4.9836	3.7342
RMSE	5.9634	6.0232	5.3427

**Table 3:** Error evaluation indicator of the prediction results of SO_2_.

Data Subsets	Error Indicator	BP	ARIMA-FC	NARX-WS	NARX-FC
First group	MAE	10.5835	9.0415	16.9133	7.1388
	RMSE	14.5723	12.8278	18.9587	8.6520
Second group	MAE	9.2676	8.4514	7.3071	5.3797
	RMSE	11.2321	10.9401	8.9681	6.1651
